# Supplementary material for: ‘Who wants to be a teacher in Ghana?’ A structural equation modelling analysis of prospective language teachers’ behavioural intentions to pursue a career in teaching
Source: PLoS One. 2025 May 8;20(5):e0323131. doi: 10.1371/journal.pone.0323131 (PMC12061143; doi:10.1371/journal.pone.0323131)
Supplement: S1 — Questionnaire.docx. (DOCX) [file pone.0323131.s001.docx]

**Introduction**

You are invited to participate in a survey aimed to investigate prospective language teachers’ willingness to become professional teachers after completion of their university education. Your sincerity is much appreciated

**SECTION A: Demographic Information**

Gender: Male [ ] Female [ ]

Age Range: 10-19 [ ] 20-29 [ ] 30-39 [ ] Above 40 [ ]

Year Group: 1^st^ Year [ ] 2^nd^ Year [ ] 3^rd^ Year [ ] 4^th^ Year [ ]

**SECTION B**

Express your opinion on the following issues about teaching on a scale of 1 to 5. Note 1 = **Strong Disagreement** and 5 = **Strong Agreement**

| **Attitude toward teaching** | **1** | **2** | **3** | **4** | **5** |
| --- | --- | --- | --- | --- | --- |
| Teaching is an enjoyable profession |  |  |  |  |  |
| I consider teaching a noble profession |  |  |  |  |  |
| I feel proud to be considered a teacher in Ghana |  |  |  |  |  |
| Teaching is a fulfilling and rewarding profession |  |  |  |  |  |
| I think teaching is a highly respected profession |  |  |  |  |  |
| The teaching profession is seen as prestigious and admirable by the general public. |  |  |  |  |  |
| I find joy in helping students learn and succeed. |  |  |  |  |  |
| I am passionate about my subject matter and enjoy sharing my knowledge with students. |  |  |  |  |  |
| **Subjective Norms** | **1** | **2** | **3** | **4** | **5** |
| my parents approve of my desire to become a teacher |  |  |  |  |  |
| my colleagues advise me to become a teacher |  |  |  |  |  |
| People I value consider teaching a good career |  |  |  |  |  |
| Ghanaians value teaching as a professional career |  |  |  |  |  |
| My parents would be proud of me if I become a professional teach |  |  |  |  |  |
| Ghanaians respect teachers a lot |  |  |  |  |  |
| People in society show genuine appreciation for the teaching profession. |  |  |  |  |  |
| **Perceived confidence related to teaching** | **1** | **2** | **3** | **4** | **5** |
| I can easily succeed as a professional teacher |  |  |  |  |  |
| Teaching would be an easier career job |  |  |  |  |  |
| I have the knowledge to become a professional teacher |  |  |  |  |  |
| I have the confidence to get students of diverse abilities to their full potentials |  |  |  |  |  |
| I can get my students to excel in school |  |  |  |  |  |
| I can get my students to easily pass exams |  |  |  |  |  |
| I think I possess the skills to make teaching successful |  |  |  |  |  |
| I consider myself a potentially competent teacher |  |  |  |  |  |
| I find controlling of students’ behaviour an easy task for me |  |  |  |  |  |
|  |  |  |  |  |  |
| **Willingness to become a professional teacher after school** | **1** | **2** | **3** | **4** | **5** |
| I am enthusiastic about pursuing a career in teaching after completing my university education. |  |  |  |  |  |
| I am motivated to overcome challenges and continuously improve as a professional teacher |  |  |  |  |  |
| Although there are other opportunities, it is my desire to become a professional teacher after my university education |  |  |  |  |  |
| I consider teaching my first-choice profession after my university education |  |  |  |  |  |
| I am always thinking of how to effectively impact students’ learning with the knowledge I acquire during my university education |  |  |  |  |  |
| It has always been my dream to contribute to society through teaching |  |  |  |  |  |
| I am willing to invest time and effort in ongoing professional development to enhance my teaching skills |  |  |  |  |  |
| I believe that education is a powerful tool for societal change, and I want to be a part of that transformation. |  |  |  |  |  |
