# Supplementary material for: ‘Who wants to be a teacher in Ghana?’ A structural equation modelling analysis of prospective language teachers’ behavioural intentions to pursue a career in teaching
Source: PLoS One. 2025 May 8;20(5):e0323131. doi: 10.1371/journal.pone.0323131 (PMC12061143; doi:10.1371/journal.pone.0323131)
Supplement: Attachment — Submitted filename: REVIEWER TEMPLETE.docx [file pone.0323131.s002.docx]

Thank you for giving the opportunity to review this manuscript 'Who Wants to be a Teacher in Ghana?' - a Structural Equation Modelling Analysis of Prospective Teachers’ Behavioural Intention to Pursue a Career in Teaching. The manuscript undoubtedly adds valuable insights into understanding the behavioural intentions of prospective teachers regarding their career choices. However, there are certain areas that require attention and refinement.

**Abstract**

The abstract contains a considerable amount of jargon. The phrase "Amidst declining interest in teaching" lacks clarity and coherence. It should be rephrased to form a complete sentence and maintain a logical flow throughout. I suggest restructuring the abstract to include sections on clarity, methodology, findings, recommendations, and policy implications. Further adjustments are recommended to enhance the abstract's effectiveness.

**Introduction**

From the review of the introduction, there are several areas that need improvement.

Weak Structure and Assumptions:

*The introduction lacks a strong structure and relies on assumptions rather than scientific evidence. For instance, statements like "This, from our perspective, has a detrimental effect..." Anecdotal evidence and popular opinions in Ghana indicate that professional teachers are always laughing stocks, as people consider the profession …………”should be revised to present evidence-based claims.

*Lack of Clear Aim and Objectives - The introduction should clearly state the aim and objectives of the study. While the motivation for the study is mentioned, it is important to outline specific research goals to guide the reader.

*Grammatical Errors: The introduction contains grammatical errors, such as awkward phrasing and incorrect use of punctuation. For example, "Several studies in Ghana, for instance, have shown that," could be revised for clarity.

*Unclear Message - The introduction fails to effectively communicate the main message to the reader. It should provide a clear overview of the study's significance, context, and objectives.

Considering these points, a thorough review and restructuring of the introduction are necessary to ensure that it meets the standards of a scientific manuscript. This process should involve revising assumptions to reflect evidence-based claims, clearly stating the aim and objectives, correcting grammatical errors, and ensuring clarity in conveying the study's message to the reader.

**2 Literature Review**

**2.1 Theoretical Framework: The Theory of Planned Behaviour**

After reading the theoretical framework, I observe some potential areas of concern:

*While manuscript attempts to explain TPB, some sentences are overly complex and can be simplified for clarity. For instance, phrases like "comprehensive perspective that has propelled the Theory of Planned Behaviour (TPB) to be at the forefront of theories frequently employed..." could be streamlined for easier comprehension.

*The discussion of theoretical framework may benefit from being more concise. Some sentences appear repetitive or overly verbose and can be condensed without losing important information.

*The flow of ideas can be enhanced to improve coherence. Transitions between the different aspects of the TPB and its components could be smoother, allowing readers to follow the discussion more easily.

*While the manuscript introduces the key concepts of TPB, ensuring accuracy in terminology and interpretation is crucial. This includes correctly representing the theoretical framework and its components, as well as accurately citing relevant literature.

I will like the authors to address these areas to improve the technical quality of the manuscript's discussion on the TPB and enhance its overall clarity and effectiveness.

**3 Methods and Materials**

**3.1 Study Design and Selection of Participants**

*Clearly describe the survey design, including its strengths and limitations.

*The authors need to provide rationale for choosing the cross-sectional descriptive survey design and justify its appropriateness for the study. The authors need explain why they adopted the cross sectional descriptive survey design

*Explain the sampling method in more detail, including how participants were selected and any potential biases.

*Grammatical Errors: The methodology contains grammatical errors, such as awkward phrasing and incorrect use of punctuation. For example, "Most importantly, we considered only the final-year prospective teachers because they had completed their teaching practicum and were aware of the experiences related to practical teaching, unlike the first-, second-, and third-year students who have not received any practical teaching experience. Thus, the final year perspective can evaluate the sense of confidence related to practical teaching, hence making them suitable candidates for this study." could be revised for clarity.

*Clarify Adaptation Process: Provide more detail on how the TPB scale was adapted for use in this study. Explain any modifications made to the original scale and justify these changes based on the specific context of the research.

*Questionnaire Structure: Describe the structure of Sections A and B of the questionnaire in more detail. Provide a brief overview of the content included in each section and explain the rationale behind this division.

*Demographic Information: Specify the demographic variables collected in Section A of the questionnaire. Include details such as age range, gender, educational background, and any other relevant demographic characteristics of the research participants.

*Likert Scale Items: Provide examples of the Likert scale items used to measure each TPB construct (behavioural beliefs, control beliefs, subjective norms, and behavioural intentions). Ensure that the wording of the items is clear, unambiguous, and aligned with the theoretical framework of the TPB.

*Pilot Testing: Discuss any pilot testing or pre-testing conducted to assess the reliability and validity of the questionnaire. Provide information on the sample size, composition of the pilot sample, and any revisions made to the questionnaire based on pilot feedback.

**Discussion of Results**

Having read the analysis and discussions, there are some suggestions for the Discussion of Results

*Clarity and Structure: The authors need to start by summarizing the main findings of the study before delving into the discussion. Also, organize the discussion into clear subsections based on the key themes or variables analyzed in the study, such as attitudes towards teaching, subjective norms, and behavioural control (Optional).

*Integration of Literature: The authors need to integrate the findings of the study with existing literature to provide context and support for the interpretations made. Ensure that each point made in the discussion is supported by references to relevant scholarly sources, such as studies on teachers' professional identity and attitudes towards teaching in Ghana. Furthermore, the authors should provide a critical analysis of the findings, acknowledging any limitations or potential biases in the data.

*Interpretation of Findings: the authors should interpret the findings in relation to the theoretical framework (e.g., Theory of Planned Behavior) and discuss how they contribute to our understanding of the topic.

*The authors should avoid overgeneralizing or drawing unwarranted conclusions from the data. Instead, focus on providing a nuanced interpretation based on the evidence presented.

*Language and Style: the authors should ensure that the language used is clear, concise, and free of jargon. Aim for a formal academic tone that is accessible to readers. Also, review the writing for grammar and punctuation errors to enhance readability and professionalism.

**Managerial Implications**

The authors should provide specific recommendations for educational institutions and policymakers on how to launch campaigns and initiatives aimed at reshaping societal attitudes towards teaching. Clarify how showcasing successful teachers, improving working conditions, and emphasizing the role of teachers can be implemented in practice. Also, alternative strategies or modifications to the recommendations that may be more feasible in the context of educational institutions and policy-making.

*Provide evidence to support the recommendations from research or best practices in the field. Provide examples of successful campaigns or initiatives that have effectively addressed negative perceptions of teaching and enhanced the profession's status and reference to relevant literature or case studies to bolster the credibility of the proposed managerial implications.

* The authors should ensure that the recommendations address the multifaceted nature of the issues and consider the potential long-term impact of the proposed recommendations on teacher recruitment, retention, and overall morale.

Overall, in the review of the manuscript, several areas for improvement were identified across different sections. Starting with the Introduction, reviewers noted that the section lacked a clear structure and failed to effectively convey the study's purpose and significance. I recommended revising the introduction to provide a clearer overview of the research aims and objectives, as well as to establish the context for the study more effectively. In the Literature Review, reviewers suggested strengthening the integration of existing literature to provide more robust theoretical support for the study. I advised ensuring that the review is comprehensive and well-organized, with a clear progression of ideas and a critical analysis of relevant studies.

Moving on to the Abstract, i notice that while it provided a brief summary of the study, it lacked clarity and did not follow a structured format. I recommended restructuring the abstract to include sections on clarity, methodology, findings, recommendations, and policy implications, and ensuring that each section flows logically and succinctly summarizes the key points of the study. Overall, the review aimed to provide constructive feedback to enhance the clarity, coherence, and overall effectiveness of the manuscript, from the Introduction and Literature Review to the Abstract and beyond.
