# Supplementary material for: ‘Who wants to be a teacher in Ghana?’ A structural equation modelling analysis of prospective language teachers’ behavioural intentions to pursue a career in teaching
Source: PLoS One. 2025 May 8;20(5):e0323131. doi: 10.1371/journal.pone.0323131 (PMC12061143; doi:10.1371/journal.pone.0323131)
Supplement: Attachment — Submitted filename: Response to Reviewers.docx [file pone.0323131.s003.docx]

# Rebuttal Letter

# Abstract

**Comment:** Moving on to the Abstract, I notice that while it provided a brief summary of the study, it lacked clarity and did not follow a structured format. I recommended restructuring the abstract to include sections on clarity, methodology, findings, recommendations, and policy implications, and ensuring that each section flows logically and succinctly summarizes the key points of the study. Overall, the review aimed to provide constructive feedback to enhance the clarity, coherence, and overall effectiveness of the manuscript, from the Introduction and Literature Review to the Abstract and beyond.

**Response:** Thank you for the suggestion. The suggestion has been taking into consideration as suggested

**Comment:** The abstract contains a considerable amount of jargon. The phrase "Amidst declining interest in teaching" lacks clarity and coherence. It should be rephrased to form a complete sentence and maintain a logical flow throughout. I suggest restructuring the abstract to include sections on clarity, methodology, findings, recommendations, and policy implications. Further adjustments are recommended to enhance the abstract's effectiveness.

**Response:** Thank you for the suggestion. The suggestion has been taking into consideration as suggested

.

# Introduction

**Comment 1:** Reviewer #1: Overall, in the review of the manuscript, several areas for improvement were identified across different sections. Starting with the Introduction, reviewers noted that the section lacked a clear structure and failed to effectively convey the study's purpose and significance. I recommended revising the introduction to provide a clearer overview of the research aims and objectives, as well as to establish the context for the study more effectively. In the Literature Review, reviewers suggested strengthening the integration of existing literature to provide more robust theoretical support for the study. I advised ensuring that the review is comprehensive and well-organized, with a clear progression of ideas and a critical analysis of relevant studies

**Response:** As could be seen in pages 3 and 4, we had already clearly outlined the purpose of the as follows:

*“Thus, the motivation for the current study is to investigate the willingness of prospective teachers to become professional teachers. We particularly seek to do this investigation using the theory of planned behaviour (TPB) as a theoretical lens. Using the TPB, we specifically seek to investigate how (1) prospective teachers’ attitudes (behavioural beliefs) towards teaching influence their willingness to become professional teachers, (2) how social pressure and approvals of significant others (subjective norms) influence prospective teachers’ willingness to become professional teachers, and (3) how the sense of efficacy in teaching (control beliefs) influence their willingness to become professional teachers.”* We therefore wonder what other purpose of the study the reviewer expects us to clearly outline. We would be glad if the reviewer could be more specific

**Comment 2: Reviewer 2#** However, the contributions of the study are not stated in the introduction. Even though, I expect elaborate discussion of contributions in the literature review section, brief highlight of contributions in the introduction is also useful for readers to know the contribution of a study right from its introduction section.

**Response:** We thank the reviewer for this contribution. However, from our standpoint, the discussion of results and implications of the study sections are the ideal sections for demonstrating how a study contributes to readers understanding of a particular topic. We do not intend to nullify the significance of the reviewer’s submission; nonetheless, per our writing style, dedicating the entire introduction to background issues that compels readers to find out what the study seeks to achieve is ideal. However, if the journal requires that the contributions of the study set the tone in the introduction, we would be ready to take that into consideration..

**Comment 3: Reviewer 2#** Moreover, the authors do not present any peer-reviewed or published evidence/argument(s) in support of the claim that “teaching as a professional career is persistently declining in its value as a reputable career opportunity in Ghana and other African countries alike”. I encourage authors to do this to give more structural credibility to the content of the introduction in terms of the authenticity and severity of the problem being examined. Remember to support all arguments with statistics and/or literature where necessary and available.

**Response:** The suggestion above has been addressed. We have provided a citation about the declining status of the teaching profession in Ghana and other African countries, as can be observed on page 3, paragraph 2.

**Comment 4:** Reviewer #3: The introduction is poorly written. If you begin on the premise, what is the essence of your study? Better framing would have been using data on teacher attrition and teacher development in Ghana to justify your study. Then offer solid rational for studying teacher motivation.

**Response:** We thank the reviewer for this suggestion. We have addressed this issue. With relevant literature, we have grounded the study on the premise that teacher attrition in Ghana is high. Moreover, the value of the teaching profession is said to be declining, according to literature. This thus made us wonder whether prospective teachers would develop a favourable behaviour

**Response:**

**Comment 5:** If they already in teacher training programme, then they have already made their mind or decided their career choice.

**Response:** We thank the reviewer for this submission. However, we wish to express our disagreement with this fact. The fact that these students are in the teacher training programme does not guarantee that they have made their career choices already. Theoretically, that is the expectation. However, this deviates from reality because my interactions with the students I teach in higher education in Ghana show that most of them are contemplating their career choices given the economic conditions of the country and the reputation regarding the economic condition of the profession. Moreover, taking a pursuing profession course in teaching in Ghana does not mean that graduates are limited to teaching only; they equally have the opportunity to work in other sectors of the economy as well.

Moreover, all students are, at the end of the end of their final year, given entrepreneurial education. This initiative suggests that all graduates are encouraged to venture into entrepreneurship. This also buttresses the fact that students in the context of this study are encouraged to seek other employment opportunities despite being professionally trained as teachers.

**Comment 6:** Current status of teachers in Ghana could also help in justifying why we need to study teacher motivation.

**Response:** This is already captured in the introduction. The introduction is emphatic on the fact that teachers’ status is quite unfavourable given the declining teacher reputation and the constant rant about bad conditions of service, which consequently affect teacher retention and commitment to the profession (see pages 2 and 3).

**Comment 7: ***The introduction lacks a strong structure and relies on assumptions rather than scientific evidence. For instance, statements like &quot; This, from our perspective, has a detrimental effect...& quot; Anecdotal evidence and popular opinions in Ghana indicate that professional teachers are always laughing stocks, as people consider the profession …………” should be revised to present evidence-based claims.

**Response:**  We thank the reviewer for pointing out this issue. We have addressed this issue by adding more empirical literature that supports our assumptions, as could be observed in the introduction.

Comment: The introduction contains grammatical errors, such as awkward phrasing and incorrect use of punctuation. For example, &quot; Several studies in Ghana, for instance, have shown that, & quot; could be revised for clarity**.**

**Response:** the said correction has been made, as could be seen in the first paragraph of the introduction

**Comment. *******Unclear Message - The introduction fails to effectively communicate the main message to the reader. It should provide a clear overview of the study's significance, context, and objectives.

**Response**: We thank the reviewer for this suggest. However, the suggestion is quite unclear, since we have already given the overview of the study as well as the objectives of the study

# Literature review

**Comment 1:** Excellent empirical literature review. However, it should be strengthened in the following ways:

i. The authors must highlight the salient gaps in the literature and demonstrate how they intend to contribute to filling them.

**Response:** This has been addressed. After the discourse in the introduction, we stated as follows:

Apart from the potential repercussion of in-service teachers' unfavourable evaluation of the teaching profession (1) on their instructional engagements and students’ academic excellence, we assume in the context of this study that such evaluations and the reputation surrounding the professional identity of teachers could affect the willingness of prospective teachers to become professional teachers after their professional teacher education programmes in higher education. This appears to be a gap in the literature yet to receive enough scholarly attention. Thus, the current study aims to fill this scholarly void by investigating the willingness of prospective teachers to become professional teachers. We particularly seek to do this investigation using the theory of planned behaviour (TPB) as a theoretical lens (see second paragraph on page ).

**Comment 2:**  The authors must review a few more current empirical literatures on the specific subject of “Prospective Teachers’ Behavioral Intention to Pursue a Career in Teaching”. Even if you don’t literature related to teaching you could find some related to other professions/careers.

**Response:** This has been done already, as could be seen in the conceptual model section.

# Theoretical framework

**Comment 1: Reviewer #3.** TPB has been applied in studies in Ghana and thus, the authors should do a job in explaining how the tenets were operationalized for their study. Explain what the theory is about then indicate how you are going to operationalize it in your study

**Response:** We thank the reviewer for this suggestion aimed at improving the overall quality of the paper. However, this suggestion has already been dealt with under the theoretical framework and model conceptualization sections. The theoretical framework section was particularly dedicated to defining Ajzen’s TPB and its tenets. We discussed how the TPB, according to Ajzen, could be applied to understand the forces behind individuals’ actions. Under the conceptual model section, we moved on to meticulously present and discuss the TPB theory, which was used to measure prospective teachers’ behaviour regarding their willingness to pursue a career in teaching. As we clearly stated in the opening paragraph of the conceptual model section:

“Drawing on the theory of planned behaviour, our current study aims to investigate how prospective teachers’ behavioural beliefs about professional teaching, the subjective norms about professional teaching, as well as their control beliefs, significantly contribute to their behavioural intention to become professional teachers after their teacher training education at the university. It is, thus, considered a necessity to conceptualise all these constructs, taking into consideration current research findings on how these constructs predict individuals’ actions in various domains.”

Having clearly defined how the TPB was to be applied in the current study, we find it quite challenging to understand what else the reviewer expects in terms of “operationalizing the theory” in our study. All our operational hypotheses were based solely on the tenets of the theoretical framework and empirical literature. However, we would be glad if the reviewer could be more specific on the exact thing we should do that is different from what we have already done.

**Comment 2:** While manuscript attempts to explain TPB, some sentences are overly complex and can be simplified for clarity. For instance, phrases like "comprehensive perspective that has propelled the Theory of Planned Behaviour (TPB) to be at the forefront of theories frequently employed..." could be streamlined for easier comprehension.

**Response**: the entire section has been language-edited to improve clarity and comprehensibility. We thank the reviewer for pointing this out

**Comment 3**: The discussion of theoretical framework may benefit from being more concise. Some sentences appear repetitive or overly verbose and can be condensed without losing important information.

**Response:** this has been addressed (refer to response to comment 2)

**Comment**: The flow of ideas can be enhanced to improve coherence. Transitions between the different aspects of the TPB and its components could be smoother, allowing readers to follow the discussion more easily.

**Response:** Addressed

# Conceptual model

**Comment: Reviewer #3.** Section 2.2 could be deleted. If you explain your model properly, you could highlight how it has been applied then come up with your hypothesis. Ajzen has already argued that the three predictors mediate through intention to predict a given behaviour. The studies you used to developed your hypothesis has nothing to do with teacher motivation! All that you hypothesized was advanced by Ajzen!

**Response:** We thank the reviewer for the suggestion. However, we wish to oppose this suggestion. Foremost, we wish to declare that there is a need to maintain the conceptual model. In applying PLS-SEM, there is a need to explain the theoretical framework that underlies the study. However, there is always a need to build a model out of the theoretical framework to show how the theory is practically used in the study. As we did in the study, we did not use all the tenets of the TPB. Thus, there is a need to adapt the TPB in a conceptual form to show how it is applied.

Also, there seems to be no established convention that the literature reviewed in a particular study must be exactly related to the study’s topic. Thus, stating that the reviews have nothing to do with teacher motivation is unacceptable from our standpoint. The main aim of the reviews under the conceptual model was to revisit studies that have used some of the tenets of the TPB to understand human behaviour. Besides, the issues discussed are within the scope of teachers and school-related issues. It does not deviate significantly from the study’s topic. Moreover, we wish to declare that the study is not even about teacher motivation; it is rather about their intention—from our perspective, intention and motivation are not the same.

# Methodology

**Comment: Reviewer 2#**The methodology deployed seems inadequate as some important information regarding sampling, sample size etc. are missing.

**Response:** This has been resolved, the population and sample are been clearly defined and justified

**Comment: Reviewer 2#** It is not clear why you chose only two schools and not say five, or ten schools. Even if the two schools chosen is enough, why would you choose say school A and B instead of C and D? While you may not have to mention the names of the schools, their selection criteria must be revealed to inform the validity of the sample. Similarly, why did you choose to focus on Ghanaian languages and not core subjects? In a nutshell, you must provide a scientific selection criterion rather than a personal or arbitrary selection criteria.

**Response:** We have justified our selection criteria. We must state that our study focused solely on language teachers in two higher education institutions that specialise in training indigenous language teachers. For this reason, we have modified our research topic to limit the generality of the study. (See page 9)

**Comment: Reviewer 2#** The Again, the sampling technique employed in selecting the 111 respondents is not mentioned, let alone what led to the decision to use 111 respondents. All these must be discussed in the revised version of the manuscript. I understand that you have included some of them as limitations of the study, but you still have to explain your choices because knowing the selection/choice criteria give readers a good understanding of how scientific are the methodological procedures you deployed for the study.

**Response:** We had already stated that the census method, which involves obtaining data from all suitable participants, was used for the selection of participants. We have now indicated in the manuscript that, despite our intention to get data from all 195 prospective language teachers, only 111 filled out the questionnaire administered. Nonetheless, per the sample size determination criteria offered by Krecjie and Morgan, 111 is representative of 195 and could therefore aid in the in the generalisation of findings.

**Comment: Reviewer 3#** The method tells me that the who study frame is misleading. If you drew on language teachers, then that should drive your who study… why did recruit language teachers? You did not study teachers’ intention however; you studied language teachers’ intention.

**Response:** Surely, we agree with this submission. Consequently, we have modified the topic of the study from prospective teachers to prospective language teachers. This was done to clear up any confusion regarding the scope of the study, as the reviewer pointed out.

**Comment: Reviewer#** There is lack of transparency regarding how the instruments were developed. How were they developed? How can we trust the content and its alignment to the tenets of the TPB? We cannot trust the result until this is substantiated.

**Response:** We have provided further explanation for how the instrument’s content validity was ensured. Moreover, to give the reviewer an idea of the structure of the questionnaire, we have uploaded the questionnaire for review.

# Results

**Comment:** the discussion could be strengthened by highlighting some plausible explanation why, for instance, subjective norms about teaching influences entry into the teaching profession. I urge authors to do the same for the remaining two factors of TPB (i.e., behavioral control— and behavioral beliefs)

**Response:** Thank you to the reviewer for this suggestion. However, we are certain that the current discussion is primarily focused on explaining why these factors significantly influence teachers behavioural intentions. This was done with particular reference to existing literature.

# Implications of the Study

**Comment:**  authors could have said something about the extent to which your findings can be generalized and highlighted contexts in which they could be applicable.

**Response:** We thank the reviewer for this suggestion. However, throughout the discussion, we have highlighted how the findings could have implications for teacher commitment and practices across the nation.

# Formatting

**Comment:** The authors should check the journal’s guidelines and format their paper according. E.g., abstract and referencing

**Response: This has been addressed**
