# Supplementary material for: ‘Who wants to be a teacher in Ghana?’ A structural equation modelling analysis of prospective language teachers’ behavioural intentions to pursue a career in teaching
Source: PLoS One. 2025 May 8;20(5):e0323131. doi: 10.1371/journal.pone.0323131 (PMC12061143; doi:10.1371/journal.pone.0323131)
Supplement: Attachment — Submitted filename: Response_to_Reviewers_auresp_4.docx [file pone.0323131.s006.docx]

**Response to Reviewers**

Reviewer #1: I have read the reviewers' comments and authors feedback. It is important that the authors carefully review the comments and answers the questions accordingly. Instead of the authors justifying the comments, they need to correct and modify the manuscript. Additionally, I still have concerns regarding the grammar, which does not meet the required technical standards of the journal. Thanks

Response: the grammatical issues has been taking care of. Thank You

Reviewer #2: PONE

1. Some proofreading is still required to fix a few errors.

Response: the manuscript has been proofread.

2. Introduction
   1. Authors seems to be unsure of their contributions because they claim that “This appears to be a gap in the literature yet to receive enough scholarly attention. Gaps must be real, factual and contributions should be obvious and relevant coming directly from the identified gap(s).

**Response:** I seem the reviewer pointed this out because of the choice of construction “This appears...” to make this clear we have rephrased the entire section making our gap emphatic as could be observed in page 4 paragraph 2

3. Literature review
   1. Excellent empirical literature review. However, it should be strengthened in the following ways:

a. The authors must highlight the salient gaps in the literature and demonstrate how they intend to contribute to filling them.

**Response:** We have provided a new subsection that reviews empirical studies related to student-teachers' behavioural intentions towards pursuing a career in teaching (see page 9 and 10). At the end of the section, we highlight an empirical gap the study fills.

b. The authors must review a few more current empirical literature on the specific subject of “Prospective Teachers’ Behavioral Intention to Pursue a Career in Teaching”. Even if you don’t literature related to teaching you could find some related to other professions/careers.

**Response:** Refer to response to (a). The new subsection added recent literature to the topic

4. Methods and Materials
   1. So, what was the sampling technique? Random sampling or a mere point of saturation was employed?

**Response:** In our methods section, we stated emphatically that the we used census method (which involves a consideration of all possible research subjects in an investigation). Hence, it is difficult to understanding why the reviewer expects us to state whether we did simple random sampling or not. We stand to be corrected anyway, but we are certain that census method is another procedure used in getting research subjects for an investigation. But considering all subjects does not necessary all of the population would be involved. As could be observed in our study, the decision was to include all members of the population, by only 180 of them respondent to the questionnaire. It was a mere point of saturation either. The sampling technique is census method (census sampling). This is one of the many studies that employed this sampling technique:

BISWAS, S., & SEHGAL, V. K. (1987). Generalized probability model for estimating the proportion of the female population at different levels of fertility based on census sampling from a mixed population. *International Journal of Systems Science*, *18*(10), 1909–1917. <https://doi.org/10.1080/00207728708967163> .

Janatolmakan, M., Andaieshgar, B., Aryan, A., Jafari, F., & Khatony, A. (2019). Comparison of Depression Rate Between the First- and Final-Year Nursing Students in Kermanshah, Iran. *Psychology Research and Behavior Management*, *12*, 1147–1153. <https://doi.org/10.2147/PRBM.S238873>

Raige, S. R., Stellefson, M., Chaney, B. H., & Alber, J. M. (2015). Pinterest as a Resource for Health Information on Chronic Obstructive Pulmonary Disease (COPD): A Social Media Content Analysis. *American Journal of Health Education*, *46*(4), 241–251. https://doi.org/10.1080/19325037.2015.1044586

5. Results
   1. The discussion could be strengthened by highlighting some plausible explanation why, for instance, subjective norms about teaching influences entry into the teaching profession. I urge authors to do the same for the remaining two factors of TPB (i.e., behavioural control— and behavioural beliefs)

**Response:** We would like to urge the reviewer to critically have a second look at the discussion section. Our discussion touches on why the three predictors had a significant association with Intention as well as the possible as the possible implications. Probably, the reviewer does not appreciate the style in which the arguments were presented. We have highlighted those areas in red.

6. Implications
   1. Authors could have said something about the extent to which your findings can be generalized and highlighted contexts in which they could be applicable.

**Response:** We have addressed this issue. Under the managerial implication we have identified specific context the findings could be applied. We have stated that: “Though the study was conducted in a specific context of language teaching, which does not fully represent the broader context of teachers in other academic fields, the findings of the current study have implications for teachers in general. It provides insight into the career intention of prospective teachers in other fields, given that teachers' working conditions in Ghana, irrespective of the field of the teacher, are perceived to be suboptimal. The findings also imply any context where the profession suffers from a poor reputation as well as in contexts like Ghana African countries alike where the government seeks to improve teacher recruitment and retention.”
